# Enhancer of zeste homologue 2 (EZH2) is a reliable immunohistochemical marker to differentiate malignant and benign hepatic tumors

**DOI:** 10.1186/1746-1596-7-86

**Published:** 2012-07-18

**Authors:** Szofia Hajósi-Kalcakosz, Katalin Dezső, Edina Bugyik, Csaba Bödör, Sándor Paku, Zoltán Pávai, Judit Halász, Krisztina Schlachter, Zsuzsa Schaff, Péter Nagy

**Affiliations:** 1 First Department of Pathology and Experimental Cancer Research, Semmelweis University, Üllõi út 26, Budapest, H-1085, Hungary; 2 Department of Anatomy and Embriology, University of Medicine and Pharmacy, Targu Mures, Romania; 3 Second Department of Pathology, Semmelweis University, Budapest, Hungary; 4 Tumor Progression Research Group, Joint Research Organization of the Hungarian Academy of Sciences and Semmelweis University, Budapest, Hungary

**Keywords:** Immunohistochemistry, EZH2, Hepatocellular carcinoma, Cholangiocarcinoma, Hepatoblastoma, Metastasis, Hepatocellular adenoma

## Abstract

**Background:**

The immunohistochemical demonstration of Enhancer of zeste homologue 2 (EZH2) proved to be a useful marker in several tumor types. It has been described to distinguish reliably hepatocellular carcinomas from liver adenomas and other benign hepatocellular lesions. However, no other types of malignant liver tumors were studied so far.

**Methods:**

To evaluate the diagnostic value of this protein in hepatic tumors we have investigated the presence of EZH2 by immunohistochemistry in hepatocellular carcinomas and other common hepatic tumors.

EZH2 expression was examined in 44 hepatocellular carcinomas, 23 cholangiocarcinomas, 31 hepatoblastomas, 16 other childhood tumor types (rhabdomyosarcoma, neuroblastoma, Wilms’ tumor and rhabdoid tumor), 17 metastatic liver tumors 24 hepatocellular adenomas, 15 high grade dysplastic nodules, 3 biliary cystadenomas, 3 biliary hamartomas and 3 Caroli’s diseases.

**Results:**

Most of the malignant liver tumors were positive for EZH2, but neither of the adenomas, cirrhotic/dysplastic nodules, reactive and hamartomatous biliary ductules stained positively.

**Conclusions:**

Our immunostainings confirm that EZH2 is a sensitive marker of hepatocellular carcinoma, but its specificity is very low, since almost all the investigated malignant liver tumors were positive regardless of their histogenesis. Based on these results EZH2 is a sensitive marker of malignancy in hepatic tumors. In routine surgical pathology EZH2 could be most helpful to diagnose cholangiocarcinomas, because as far as we know this is the first marker to distinguish transformed and reactive biliary structures. Although hepatoblastomas also express EZH2, the diagnostic significance of this observation seems to be quite limited whereas, the structurally similar, other blastic childhood tumors are also positive.

**Virtual Slides:**

The virtual slide(s) for this article can be found here: http://www.diagnosticpathology.diagnomx.eu/vs/1173195902735693

## Background

The value of correct and reproducible classification of cancers is increasing due to the more specific, occasionally personalized therapeutic choices. Often, the traditional hematoxylin and eosin (H&E) stained sections do not provide enough information to make an adequate therapeutic decision. Immunohistochemistry is still the most widely used ancillary technique. Most of the applied antigens/antibodies can be divided into three groups. In the biggest group, there are antigens which are specific for cell types and they give information about the histogenesis of tumors e.g. thyroid transcription factor-1 (TTF-1), prostate specific antigen (PSA). In the second group, there are antigens which help to make a distinction between malignant and benign neoplasms e.g. glypican 3, P53, galectin-3. The third rapidly expanding group provides predictive information if the potential target molecule of the therapy is present on the examined tumor sample e.g. hormone receptors, Her-2, epidermal growth factor receptor (EGFR). Therefore, the establishment of a clinically valuable diagnosis often requires the application a battery of antibodies. Especially, when accurate diagnosis is expected from smaller and earlier lesions the characterization and application of novel antibodies is necessary.

Pathologists are often faced with similar problem with specimens derived from hepatic tumors or tumor like lesions. Hepatocellular carcinoma (HCC) is the most common type of primary malignant liver tumor. Less differentiated HCCs can be mistaken for biliary or metastatic carcinomas. It also can be challenging, especially in case of highly differentiated tumors, to distinguish these from dysplastic nodules or hepatocellular adenomas. Hepatocyte paraffin (Hep Par)-1 and CD 10 antibodies stain hepatocytes and hepatocyte derived tumors in paraffin embedded tissue [1,2]. Thus, they help to identify the hepatocytic origin of a tumor but do not reflect whether it is benign or malignant. In addition, they may be negative in poorly differentiated HCC [3]. Glypican-3, alpha-fetoprotein (AFP), heat shock protein 70 (Hsp70) glutamine synthetase, clathrin heavy chain [3-7] antibodies are reported to be distinctive for HCC, but none of these antibodies are flawless. They are occasionally positive in non malignant liver or in non HCC tumors [8]. Delta like protein (DLK) is a new sensitive and specific marker, which can be used together with AFP to diagnose hepatoblastoma [9]. Cholangiocarcinomas, (CC) also pose difficulties in diagnosis. They are usually adenocarcinomas and therefore their distinction from metastatic tumors or sometimes from HCC is a common problem. It can also be difficult to distinguish highly differentiated cholangiocarcinomas from ductular reactions or biliary hamartomas. Recently αvβ6 integrin has been reported to be a highly specific imunohistochemical marker for cholangiocarcinoma [10] but this antigen is also present in reactive biliary proliferations. In summary, the panel of antibodies available for immunohistochemical diagnosis of hepatic tumors has expanded substantially in the last few years. Due to focal staining patterns and cross-reactions with other tissues, diagnostic difficulties are still commonly encountered in this field, and form the basis of the ongoing search for newer and better immunomarkers.

Recently, enhancer of zeste homologue 2 (EZH2), a new marker for hepatocellular carcinomas has been described [11]. EZH2 is the catalytic subunit of polycomb repressive complex 2 (PRC2). It catalyzes trimethylation of lysine 27 on histone H3 (H3K27me3) and mediates transcriptional silencing [11]. Also, EZH2 plays an important role in the maintenance of the proliferative and self-renewal capacity of hepatic stem/progenitor cells and their differentiation [12,13]. Cai et al. [11] reported that this new marker was able to distinguish HCCs with high accuracy from hepatocellular adenomas, focal nodular hyperplasias (FNH) and dysplasic nodules. However, no other malignant liver tumors were analyzed in this study. EZH2 has been detected in tumors with various origins, such as urothelial carcinoma, squamous cell carcinoma of the esophagus, gastric cancer, glioma, renal cell carcinoma, non-small cell lung carcinoma (NSCLC), colorectal carcinoma and breast cancer [14-21]. Therefore, we decided to examine the expression of EZH2 by immunohistochemistry in various histological types of hepatic tumors and tumor-like lesions to investigate its discriminatory diagnostic value. EZH2 staining proved to be positive in most of the malignant liver tumors regardless of their origin. Thus we can confirm the result of Cai et al. [11] that EZH2 is a sensitive marker of hepatocellular carcinomas. However, its specificity is very low, this antibody does not help to distinguish histogenesis of different hepatic malignancies, since all the common types of liver cancer stain positively for this marker.

## Methods

We selected 44 HCCs, 23 CCs, 31 hepatoblastomas, 16 other childhood tumors (rhabdomyosarcomas, neuroblastomas, Wilms’ tumors, rhabdoid tumors), 17 metastases, 24 hepatocellular-, 3 biliary adenomas, and 6 ductal plate malformations (3 biliary microhamartomas and 3 Caroli’s disease), 15 high grade dysplastic nodules from the archives of the I^st^ and the II^nd^ Departments of Pathology, Semmelweis University (Budapest, Hungary). Their clinico-pathological characteristics are summarized in Table 1. The HCCs were graded according to Edmondson and Steiner [22], the CCs were classified into 3 grades [10]. The study was approved by the ethical committee of Semmelweis University.

**Table 1:** Patients’ characteristics

	**Hepatocellular carcinoma (n = 44)**		**Cholangiocarcinoma (n = 23)**	
Age (mean)	58		58	
Age (range)	42-85		30-80	
Gender (male/female)	32/12		13/10	
Histological grading	Core/surgical		Core/surgical	
I.	12	8/4	5	0/5
II.	22	10/12	14	7/7
III.	9	6/3	4	3/1
IV.	1	0/1		
Other tumors				
Hepatoblastoma	n = 31	2/29		
Hepatocellular adenoma	n = 24	3/21		
Biliary cystadenoma	n = 3	0/3		
Metastatic tumor	n = 17	9/8	8 colon, 2 breast, 2lung, 3neuroendocrine, 1 urothel, 1pancreas	
Childhood tumors	n = 16	0/16	4 rhabdomyosarcoma, 5neuroblastoma, 5 Wilms’ tumor, 2 rhabdoid tumor	
Non tumorous lesions				
High grade dysplastic nodule	n = 15	0/15		
Biliary hamartoma	n = 3	0/3		
Caroli’s disease	n = 3	0/3		

Formalin-fixed paraffin-embedded tissue was used for the immunohistochemical reactions. Staining was performed using an automated Leica Bond immunostainer, with the Leica Bond Polymer refine detection system and 3,3' Diaminobenzidine (DAB) as the chromogen. Antigen retrieval was achieved with Bond Epitope Retrieval Solution 2 (high pH) for 20 minutes. The primary antibody was a mouse monoclonal anti-EZH2 (clone 11/EZH2) from BD Biosciences (San Jose CA, USA) (dilution 1:100). The reaction resulted in nuclear staining. Scores were assigned based on the density of positivity by using negative (score = 0, no staining), weak (score = 1, <25% of nuclei staining), moderate (score = 2, 25-75% of nuclei staining) and strong (score = 3, >75% of nuclei staining).

Statistical analysis was performed by Fisher exact test.

## Results

### Hepatocellular tumors and tumor like lesions

**Figure 1:**
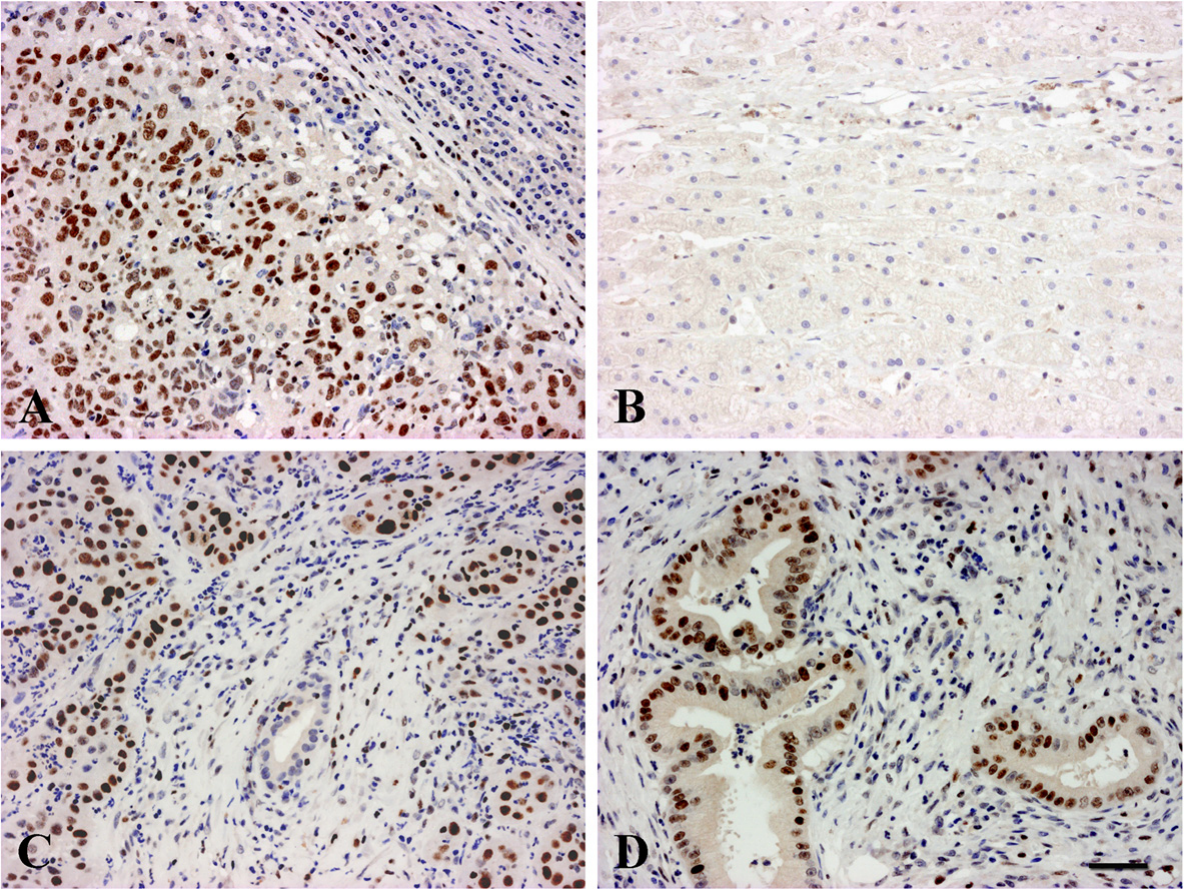
**EZH2 staining in primary liver tumors. A/HCC, nuclear staining in tumor cells, the surrounding liver is negative; B/hepatocellular adenoma, there is no staining; C/CCC, note the unstained nuclei of the non tumorous bile duct in the center¸D/positively stained highly differentiated CCC (Klatskin tumour).** Scale bar for the Figure 1:50 μm.

First we checked if we can reproduce the original observations of Cai et al. [11]. Forty of the examined HCCs (n = 44) stained positively for EZH2 (Table 2). The immunostaining always resulted in a strong nuclear reaction and the density of positive nuclei was relatively evenly distributed (Figure 1A). No correlation was found between the tumor grade, histological type and staining scores (p = 0,7972). Background staining in the surrounding parenchyma was only occasional, even at low magnification, the immunostaining usually provided clear demarcation of the tumors.

**Table 2:** EZH2 staining

**EZH2 expression**	**Negative (score0)**	**Weak (score1)**	**Moderate (score2)**	**Strong (score3)**	**Sensitivity/Specificity**
Hepatocellular carcinoma (n = 44)					0.90/0.33
Grade I.	2	6	4	0	
Grade II.	1	12	7	2	
Grade III	1	4	2	2	
Grade IV.	0	1	0	0	
CC (n = 23)					0.96/0.18
Grade I.	0	2	2	1	
Grade II.	1	2	9	2	
Grade III	0	1	3	0	
Hepatoblastoma (n = 31)	2	6	9	14	0.94/0.24
Hepatocellular adenoma (n = 24)	24	0	0	0	0/0
Biliary cystadenoma (n = 3)	3	0	0	0	0/0
Metastases					NC*
Colon (n = 8)	0	0	5	3	
Breast (n = 2)	0	0	2	0	
Lung (n = 2)	0	0	0	2	
Neuroendocrine (n = 3)	3	0	0	0	
Urothel (n = 1)				1	
Pancreas (n = 1)			1		
Childhood tumors					NC*
Rhabdomyosarcoma (n = 4)	0	1	2	1	
Neuroblastoma (n = 5)	0	0	3	2	
Wilms’ tumor (n = 5)	0	1	1	3	
Rhabdoid tumor (n = 2)	0	0	1	1	
Non tumorous lesions					
High grade dysplastic nodule (n = 15)	15	0	0	0	
Biliary hamartoma (n = 3)	3	0	0	0	
Caroli’s disease (n = 3)	3	0	0	0	

*NC not counted due to low case number.

None of the hepatocellular adenomas (n = 24) reacted with EZH2 antibody (Figure 1B), (Table 2). They have not been subclassified but were all beta-catenin negative by immunostaining. All the investigated high grade dysplastic nodules (n = 15) were negative for EZH2. None of the cirrhotic nodules in 18 tumor surrounding livers exhibited positive staining, although no macroregenerative nodule was present in them. These results are consistent with the original observations of Cai et al. [11].

### Cholangiocarcinomas, metastatic tumors and ductular reactions

**Figure 2:**
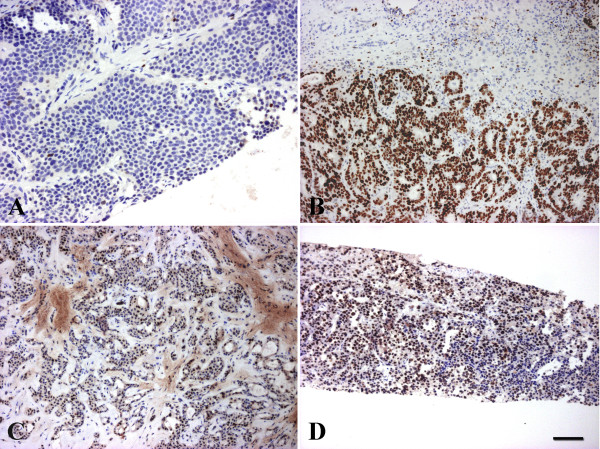
**EZH-2 staining in metastatic liver tumors. A/neuroendocrine carcinoma from the ileum is negative; while positive reaction in the nuclei of B/colon; C/breast and D/transitional cell carcinoma metastases.** Scale bar for the Figure 2: 50 μm.

Twenty-three cholangiocarcinomas were also tested, and all but one proved to be positive for EZH2. This included 3 highly differentiated, hilar cholangiocarcinomas (Klatskin tumors) (Figure 1C, D). No correlation was found between grade and the level of expression (p = 0,8051). The normal bile ducts and the reactive ductular reaction either in the peritumoral tissue or in cirrhotic livers consistently remained negative (Table 2). Three biliary cystadenomas and 6 tumor imitating ductal plate malformations (3 biliary microhamartomas and 3 Caroli’s disease) were also negative. Thus, this antibody appears to be able to distinguish neoplastic and benign or reactive biliary proliferations. Metastatic tumors present the major differential diagnostic problem for this tumor type. All the investigated metastatic adenocarcinomas (from colon, pancreas, breast and lung) were positive for EZH2 as well as the single examined transitional cell carcinoma metastasis (Figure 2). EZH2 antibody resulted in nuclear staining in these tumors as well and the distribution was mostly diffuse. Only the secondary neuroendocrine tumors (2 intestinal carcinoid tumors and 1 medullary carcinoma of the thyroid) did not exhibit positive staining.

### Hepatoblastomas and other primitive childhood tumors

**Figure 3:**
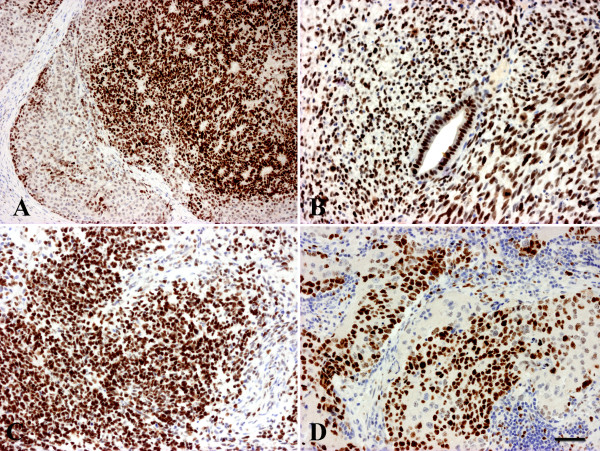
**EZH-2 staining in childhood tumors. A/hepatoblastoma, the stroma is negative, weak nuclear staining in fetal and strong reaction in the embryonal areas. B/Wilms’ tumor; C/embryonal rhabdomyosarcoma; D/neuroblastoma.** Scale bar for the Figure 3: 50 μm.

Hepatoblastoma is the most common primary malignant tumor of the liver in children. All but 2 of the investigated tumors (n = 31) were positive when staining with the EZH2 antibody (Figure 3A). The positivity was confined to the epithelial part and the staining intensity and density of the positive nuclei were usually higher in the embryonic component.

All the other investigated primitive childhood tumors (rhabdomyosarcomas, neuroblastomas, Wilms’ tumors and rhabdoid tumors) stained positively for EZH2 (Figure 3B, C, D).

## Discussion

In this study, we report that EZH2 was detected by immunohistochemistry in nearly all the investigated HCCs, CCs, hepatoblastomas, metastatic liver tumors and several other childhood cancers. However, none of the hepatocellular or biliary adenomas, high grade dysplastic or cirrhotic nodules was positive. The ductular reactions and biliary hamartomas were also consistently negative.

Knockdown of EZH2 reversed the tumorigenicity in experimental liver tumors, suggesting that EZH2 plays an important role in HCC tumorigenesis [23,24]. Increased expression of EZH2 was correlated with unfavourable outcome of HCC [25] and metastatic capacity [26]. Cai et al. [11] demonstrated convincingly that EZH2 is a highly sensitive diagnostic biomarker of HCC, which can be used to distinguish it from benign liver lesions such as hepatocellular adenomas, focal nodular hyperplasias, dysplastic and regenerative nodules. There is a complete agreement between the findings of Cai et al’s [11] and our study that all the investigated regenerative nodules and adenomas were negative. Our results also support that EZH2 is a sensitive marker of HCC. Although not reaching statistical significance, EZH2 appeared less able to recognize well differentiated HCCs in both studies. There seems to be a borderline or “grey zone” in highly differentiated hepatocellular tumors, in which EZH2 similarly to the other markers, is not absolutely reliable. It requires further direct comparative studies, which one of the recently applied markers (Glypican3, AFP, Hsp70, EZH2) is the most sensitive [3-7]. Most likely however, a panel of these antibodies would provide the most trustworthy information and EZH2 could be a useful member of this battery. All of the adenomas in our study were beta-catenin negative. The beta-catenin status of the adenomas in the study of Cai et al. [11] is not indicated. It would be of interest to examine EZH2 expression in beta-catenin positive hepatocellular adenomas, which have a higher tendency for malignant transformation [27].

In addition to hepatocellular lesions, there are other hepatic tumors, which may raise differential diagnostic problems. For this reason we investigated EZH2 expression in the most common other tumor types of liver. EZH2 staining was positive in 96% of cholangiocarcinomas, including 3 highly differentiated Klatskin tumors. As far as we know EZH2 expression has not previously been examined in this type of tumor. We have studied a few biliary cystadenomas and tumor mimicking ductal plate malformations, all of them were negative, as well as the ductular reactions. CC also must be differentiated from metastatic liver tumors. Practically all the metastatic adenocarcinomas, regardless of their origin, were positive for EZH2. Although our case number is low, the primary tumors of colon, pancreas, breast, lung [14-21] have already been described to express this tumor marker, so the few metastases we report probably do reflect reality. That is, EZH2 similarly to other CC markers e.g. CK7, CK19, claudin 4 [28] does not provide major help in distinguishing cholangiocarcinomas from metastatic tumors, but it does seem to be able to differentiate reactive/hamartomatous biliary structures and benign biliary tumors from malignant ones. This is important because so far no such marker has been reported. Even the recently described highly specific cholangiocarcinoma marker, αvβ6 integrin [10] is positive in proliferating bile ducts. The combination of these two antibodies may facilitate the diagnosis of cholangiocarcinomas.

EZH2 expression has not yet been tested on hepatoblastomas and other primitive "blastic" tumors except Ewing’s sarcoma and rhabdoid tumors which were reported positive [29,30]. The applied antibody recognizes these tumors with high sensitivity. All but two of the examined hepatoblastomas (n = 31) and the few other childhood tumors stained positively. This is not surprising, as it is considered that the major biological function of EZH2 is to maintain the undifferentiated stage of cells [31]. Again, the number of non hepatoblastomas investigated is quite low, but considering the very consistent positive staining it is highly unlikely that EZH2 could be used to differentiate among these childhood tumors. However, if recent therapeutic approaches targeting EZH2 [32] were successful, our observation in childhood tumors would gain significance.

## Conclusions

In conclusion, we can confirm the recent report of Cai et al. [11] that EZH2 is a reliable immune marker for hepatocellular carcinomas, compared to non-malignant hepatocellular lesions. EZH2 is not however, specific for HCC since almost all other examined hepatic cancers: cholangiocarcinomas, hepatoblastomas and metastatic adenocarcinomas are positive as well. Consequently, this marker does not provide help in differentiating the specific histogenesis of liver tumors, but it may well be very useful to differentiate malignant hepatocellular and cholangiocellular tumors from benign tumors and reactive lesions. As far as we know EZH2 is the first marker, which is able to do this for biliary cells derived lesions.

## Competing interests

The authors declare that they have no competing interests.

## Authors’ contributions

PN is the corresponding author and wrote the manuscript. SP and ZsS participated in study design; they coordinated and supervised the study. SzHK, KD, E.B, CsB collected the samples, they carried out part of the experiments and interpreted the data. ZP participated in the analysis and interpretation of data. JH and KS carried out the statistical analysis. All authors provided important contributions to the conception and design of the study, reviewed the results, read and approved the final manuscript.
